# Supplementary material for: Comprehensive Annotation and Functional Exploration of MicroRNAs in Lettuce
Source: Front Plant Sci. 2021 Dec 24;12:781836. doi: 10.3389/fpls.2021.781836 (PMC8739914; doi:10.3389/fpls.2021.781836)
Supplement: Supplementary file 3 [file Data_Sheet_9.ZIP › GSM2230753.plot/Lsa-miR396d_Lsat_1_v5_gn_8_152961.1_1079_TPlot.pdf]

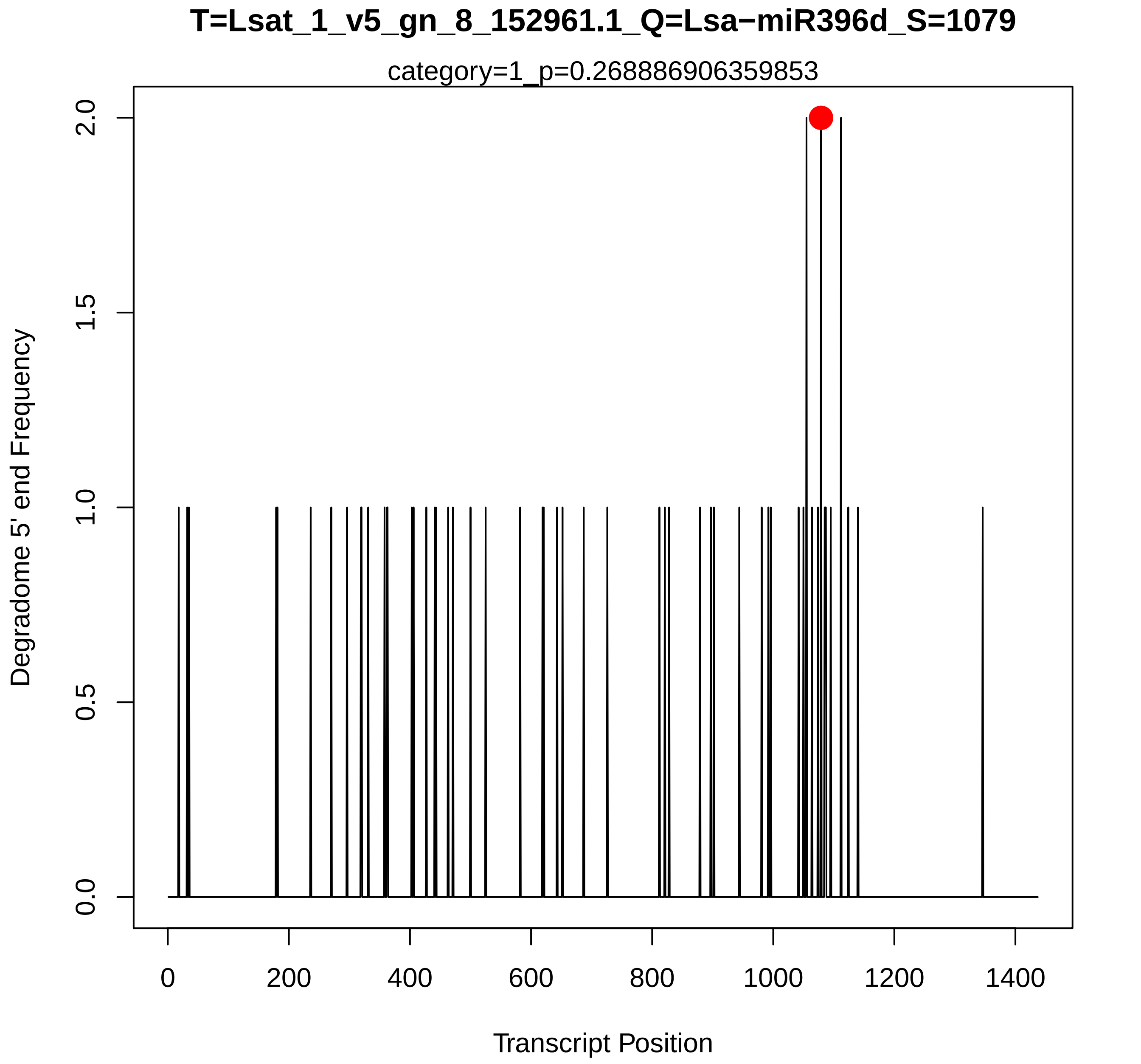
T=Lsat_1_v5_gn_8_152961.1_Q=Lsa−miR396d_S=1079
category=1_p=0.268886906359853
Degradome 5' end Frequency
0.0
0.5
1.0
1.5
2.0
0
200
400
600
800
1000
1200
1400
Transcript Position
